# An Adaptive Injection Model for Pansharpening

**DOI:** 10.1155/2023/4874974

**Published:** 2023-01-24

**Authors:** Qun Song, Chen Ding, Junhua Ren, Lili Liu, Hangyuan Lu

**Affiliations:** ^1^College of Information Engineering, Jinhua Polytechnic, Jinhua, China; ^2^Pharmaceutical College, Jinhua Polytechnic, Jinhua, China; ^3^Jiangxi Yuanda Insurance Equipment Industry Group Co., Ltd, Zhangshu, China

## Abstract

Pansharpening technology is used to acquire a multispectral image with high spatial resolution from a panchromatic (PAN) image and a multispectral (MS) image. The detail injection model is popular for its flexibility. However, the accuracy of the injection gain and the extracted details may greatly influence the quality of the pansharpened image. This paper proposes an adaptive injection model to solve these problems. For detail extraction, we present a Gaussian filter estimation algorithm by exploring the intrinsic character of the MS sensor and convolving the PAN image with the filter to adaptively optimize the details to be consistent with the character of the MS image. For the adaptive injection coefficient, we iteratively adjust the coefficient by balancing the spectral and spatial fidelity. By multiplying the optimized details and injection gain, the final HRMS is obtained with the injection model. The performance of the proposed model is analyzed and a large number of tests are carried out on various satellite datasets. Compared to some advanced pansharpening methods, the results prove that our method can achieve the best fusion quality both subjectively and objectively.

## 1. Introduction

Remote sensing aims at extracting the land information of the Earth's surface using satellites. Owing to the disadvantage of sensor technology, acquiring a high-resolution remote sensing image in both spatial and spectral domains is still difficult [1]. Therefore, image processing technology is usually adopted to fuse the existing two kinds of images, namely, the panchromatic (PAN) image which has a high spatial resolution but only one channel, and the multispectral (MS) image which has a low spatial resolution but high spectral resolution. For the MS and PAN images which are obtained simultaneously in the same area, they can be combined by appropriate algorithms to generate a fused image with high resolution in both spectral and spatial aspects. This process is also called PAN sharpening [2]. Pansharpened images are widely used in geography, military, agriculture, ecological environment, and other fields [3, 4].

The existing traditional fusion methods are usually classified into two types. The first one is component substitution (CS), which replaces the spatial components of MS images with a PAN image. Popular methods of this kind include adaptive Gram–Schmidt (GSA) [5], the principal component analysis (PCA) [6], and the intensity-hue-saturation (IHS) methods [7]. The fusion result based on the CS method has high spatial resolution but easily suffers from spectral distortion [2, 8].

The other kind is multiresolution analysis (MRA), which adds extracted details to MS images through the multiscale decomposition of PAN images. Traditional MRA methods mainly include methods based on pyramid decomposition or wavelet transform. For instance, the àtrous wavelet transform [9], discrete wavelet transform [10], and the contour wave transform [11] all belongs to this category. A nonlinear approach called morphological filters (MF) [12] has also been successfully applied in the pansharpening field. By exploring the modulation transfer function (MTF) of the MS sensor, a generalized Laplacian pyramid (GLP) based on the MTF filter; i.e., MTF-GLP [13] is presented to adaptively inject the details. To better preserve texture information in the fused image, Yang et al. [14] proposed a multiscale decomposition method based on guided filtering, and the effect is improved. Recently, the popular additive wavelet luminance proportional (AWLP) was enhanced in [15] by taking into account the acquisition device and optical radiation transfer process. The MRA method can better retain the spectral features. However, some spatial information may be lost [4, 16].

In recent years, variational optimization (VO)-based fusion methods have attracted the attention of many researchers. This kind of method usually constructs an estimation model based on the assumption of spectral and spatial fidelity between the fused image and source images. Then, a TV regularizer is proposed in [17] and applied to each band of the HRMS images. Later, Palsson et al. [18] considered a spectral reduced-rank relationship between the PAN and HRMS images and used the l2 regularizer to constrain the model. Other classes of methods such as sparse representation-based [19] and Bayesian-based [20] are also included in the VO-based methods. These methods usually can better balance the spectral and spatial quality than the traditional methods. However, they require much more computational resources [21]. Besides, the majority of the previously discussed VO methods rely on the regularization parameters, which need to be determined manually and may affect the accuracy of the model [22].

Deep learning (DL), one of the hottest research areas in artificial intelligence, has produced outstanding outcomes in the fusion of remote-sensing images. Typically DL-based networks include autoencoders (AEs) [23] and convolutional neural networks (CNNs) [24]. To better preserve spectral and spatial details, Yang et al. [16] designed an end-to-end depth residual network-based fusion method to automatically learn a mapping from remote sensing images, which has achieved good results. Recently, some unsupervised DL-based methods have been developed based on the intrinsic properties of the source images. For example, Liu et al. [25] first applied the generative adversarial network (GAN) in pansharpening and proposed PSGAN, and then Ciotola et al. [26] proposed a general unsupervised network in the full-resolution framework, thus making full use of the original information and reducing the spectral distortion. However, these methods require a lot of computation, long running time, and numerous samples [27, 28]. Owing to the limitation of the number of image samples and the requirement of algorithm efficiency, this paper comprehensively considers the abovementioned methods and optimizes the injection model based on the MRA method to achieve dual fidelity of spectral information and detailed information during fusion.

The key step in the MRA-based method is how to extract the details of the PAN image. A more advanced method is to use a linear time-invariant filter to match the point spread function (PSF) of the MS sensor. Aiazzi et al. [29] argued that it is better to use a filter to simulate the MTF of the MS sensor, but it requires using the factory information of the MS sensor, and it is not easy to obtain. Another more reliable method is to estimate the filter from the existing image to simulate the PSF of the MS sensor. In this framework, a low-spatial-resolution MS (LRMS) image can be obtained by spatially filtering a high-spatial-resolution MS (HRMS) image. The impulse response function of this filter can simulate the point spread function of the MS sensor, and the filter usually has a shape similar to the Gaussian model [30, 31]. Using this filter to convolve the PAN image, the resulting high-frequency parts are the missing detail components in the HRMS image and are highly linearly correlated with the MS image.

Considering spectral fidelity and detail injection, this work proposes a pansharpening method based on Gaussian filter estimation and adaptive detail injection. This method first obtains the initial fused image by the guided filtering injection model of multiscale decomposition, and then simulates the PSF of the MS sensor with Gaussian filtering and convolving the PAN image with the estimated Gaussian filter to obtain the optimized injected details. Then, the adaptive fusion coefficient is calculated recursively, so that the spectral fidelity and detail injection can be jointly optimal. The main innovations of our work are shown as follows:

1. An adaptive injection model is proposed to realize the dual fidelity of spectral and spatial information
2. A detail extraction method based on Gaussian filter estimation is proposed to optimize the injected details
3. An adaptive fusion coefficient is designed to automatically optimize the volume of the injected details

## 2. Related Work

### 2.1. General Framework of Injection Model

The injection model based on the MRA method refers to a fusion method in which the injection details are extracted by filtering the source images, and then the details are added to the upsampled MS image with the injection coefficients. The method can combine different pansharpening technologies according to the actual need, to exert the advantages of different fusion technologies. The general representation of the injection model is as follows [24]:

(1) $$\begin{array}{c} {\hat{MS}}_{k}={\tilde{MS}}_{k}+G_{k}*\left(P_{I}-P_{L}\right)\text{,} \end{array}$$

where *k* = 1,2,…, *N* represents the subchannel *k* in the *N* channels, ${\hat{MS}}_{k}$ represents the estimated HRMS image of the *k*-th channel, ${\tilde{MS}}_{k}$ denotes the *k*-th channel after bicubic upsampling, *G*_*k*_ represents the injection coefficient of the *k*-th channel, *P*_*I*_ represents the PAN image after histogram equalization of the *I* component of the MS image, and *P*_*L*_ represents a low-resolution version of the PAN image.

For the injection coefficient, Vivone et al. [32] propose an interchannel weight coefficient based on a regression model through an iterative algorithm. Yin and Li [33] propose a weight coefficient based on the ratio between pixel channels. Vivone et al. [34] use the high-pass modulation (HPM) method with the multiplicative combination of the source images as the injection gain. Among them, the injection coefficient using the ratio between channels can better preserve the spectral information, and the fusion result is better. The injection coefficient between channels is defined as follows:

(2) $$\begin{array}{c} G_{k}=\frac{{\tilde{MS}}_{k}}{\left(1/N\right)\sum_{i=1}^{N}{\tilde{MS}}_{k}}\text{.} \end{array}$$

### 2.2. Extraction of Intensity Component

Since it is not desirable to change the chroma and saturation while injecting details, only the intensity (*I*) component of the image is often processed in the image fusion process. The *I* component can be calculated using the following formula:

(3) $$\begin{array}{c} I=\sum_{k=1}^{N}\alpha_{k}{\tilde{MS}}_{k}\text{,} \end{array}$$

where *α*_*k*_ represents the combination coefficient of the channel. The authors in [35, 36] proposed that the intensity can be calculated using the linear combination of the bands, that is, the combination coefficients are usually acquired by solving the optimization problem as follows:

(4) $$\begin{array}{c} \min_{\alpha_{1},\ldots ,\alpha_{N}}{\left\|P-\overset{N}{\underset{i=1}{\sum }}\alpha_{i}M_{i}\right\|}^{2},s.t.\alpha_{1}\geq 0,\ldots ,\alpha_{N}\geq 0\text{,} \end{array}$$

where *P* represents the PAN image. After obtaining the *I* component of the MS image, perform histogram matching between *P* and *I* in the above formula, and obtain *P*_*I*_ using the following equation:

(5) $$\begin{array}{c} P_{I}≜\left(P-u_{P}\right)\cdot \frac{\sigma_{I}}{\sigma_{P}}+u_{I}\text{,} \end{array}$$

where *u*_*P*_ represents the mean of *P*, *u*_*I*_ represents the mean of the *I* component, *σ*_*I*_ represents the standard deviation of the *I* component; *σ*_*P*_ represents the standard deviation of *P*.

### 2.3. Guided Filtering

Guided filtering was first proposed by [24], which can save the main feature of the input image and obtain the changing trend of the guided image. Taking *P*_*I*_ as the input image and *I* as the guide image, *P*_*I*_ is generated by intensity-guided filtering to generate *P*_*L*_, and detailed information linearly related to the MS map is obtained. Guided filtering can be obtained by the following simplified formula:

(6) $$\begin{array}{c} P_{L}=GF\left(P_{I},I\right)\text{,} \end{array}$$

where GF(·) represents the guided filter function. Multiscale-guided filtering can be expressed as follows [13]:

(7) $$\begin{array}{c} P_{L}^{j}=GF\left(P_{L}^{j-1},I\right)\text{,} \end{array}$$

where *j* represents the number of guided filtering layers; *P*_*L*_^*j*^ represents the image obtained by *P*_*I*_ through the *j*-th layer of guided filtering.

## 3. The Proposed Method

### 3.1. The Proposed Framework

**Figure 1:**
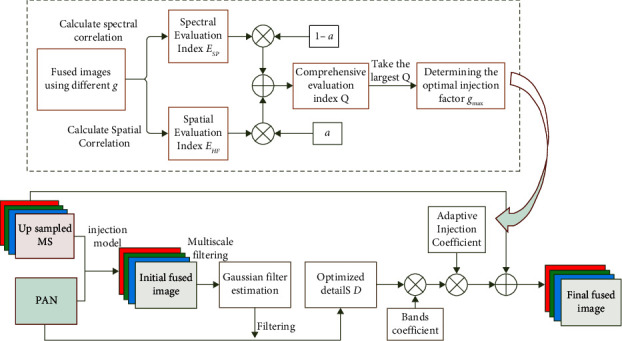
The framework of proposed fusion method.

There are two main deficiencies in the current injection model. For one thing, there is a low correlation between MS and PAN images, which may cause spectral distortion. On the other hand, there may be over-detail injection or insufficient injection. Aiming at the two problems, this work proposes a method for detail extraction and adaptive injection coefficient optimization based on Gaussian filter estimation. The framework of this method is shown in Figure 1.

Firstly, according to formula (1), the source images are used to obtain an initial fusion image through traditional injection models such as histogram matching and guided filtering.

Secondly, we proposed the detail optimization method, that is, filtering the initial fusion image with a multiscale Gaussian filter to simulate the characteristics of the MS sensor, and filtering the PAN image with the obtained Gaussian filter estimation to obtain the optimized details.

Third, we optimize the injection gain, that is, comprehensively consider the spectral information and the detail information to calculate the adaptive injection amount coefficient. The specific process is shown in the dotted box in Figure 1, and the detailed algorithm is shown in Section 3.2.

Finally, the optimized details are multiplied by the injection coefficient, and then the final HRMS image is acquired by adding the optimized details to the upsampled MS image.

### 3.2. Gaussian Filter Estimation and Detail Extraction

As mentioned above, Reference [31] proposed to use the Gaussian filter to simulate the MTF of the MS sensor; that is, the HRMS image is regarded as the LRMS image after passing through the MS sensor. A Gaussian filter is used to simulate this process, and the resulting Gaussian filter is a filtered estimate conforming to the PSF of the MS sensor. Next, the PAN image is convolved with the estimated filter, and the extracted detail components are those missing from the low-resolution MS image. Yang et al. [37] proved that the filter estimation has strong robustness through experiments. Even if white noise is added to the initial fusion image, it does not affect the extraction of details. In addition, Yin and Li [33] proposed a two-step multiscale decomposition method to refine PAN sharpening. Inspired by this, this paper proposes to use multiscale Gaussian filtering to simulate the PSF of the MS sensor, that is, extract the luminance component from the initial fusion image *F*^(1)^, denoted as *I*_1_, and perform Gaussian filtering iteratively, which can be expressed as follows:

(8) $$\begin{array}{c} I_{1}^{i}=H_{G}\left(I_{1}^{i-1}\right),i=1,2,\ldots ,n\text{,} \end{array}$$

where *I*_1_^*i*^ represents the image after the *i* th filtering and H_*G*_(·) represents the Gaussian filter. To obtain the best Gaussian filter estimation, the correlation coefficient between the result *I*_1_^*i*^ obtained by formula (8) and the *I* component of the upsampled MS image is calculated. When the correlation coefficient is the highest, the estimated Gaussian filter is the most accurate, and the correlation coefficient is calculated as follows:

(9) $$\begin{array}{c} corr\left(I_{1}^{i},I\right)=\frac{cov\left(I_{1}^{i},I\right)}{std\left(I_{1}^{i}\right)\cdot std\left(I\right)}\text{,} \end{array}$$

where cov (·) represents the covariance function and std (·) represents the standard deviation function. As the filtering level increases, the filtered image and the up-sampled MS image get closer and closer, and after reaching the maximum value, as the filtering level continues to increase, the filtered image and the upsampled MS image deviate increasingly. By iterative calculation of the values of formulas (8) and (9), the number of iterations *m* is obtained when formula (9) is maximum, which is the estimated Gaussian filter *H*_*m*_, which is expressed as follows:

(10) $$\begin{array}{c} H_{m}\left(\cdot \right)=\underset{m}{\underbrace{H_{G}\left(H_{G}\left(\cdots \right.H_{G}\left(\cdot \right)\right)}}\text{,}\\ m=\underset{i}{\arg}\max\;cor\;r\left(I_{1}^{i},I\right)\text{,} \end{array}$$

where *H*_*m*_ means the best match of the PSF of a sharpened image blurred by the MS sensor. The filter *H*_*m*_ is applied to the PAN image, and the extracted detail components should also conform to the MS sensor PSF, thus reducing the spectral distortion, and the detail extraction *D* defined as follows:

(11) $$\begin{array}{c} D=P_{I}-H_{m}\left(P_{I}\right)\text{.} \end{array}$$

Combining formulas (1), (7), and (11), after optimizing the details, the *k* th channel fusion image *F*_*k*_^(2)^ can be updated to the following form:

(12) $$\begin{array}{c} D=P_{I}-H_{m}\left(P_{I}\right)\text{.} \end{array}$$

### 3.3. Adaptive Injection Coefficient

After obtaining the optimized injection details, this paper optimizes the injection coefficient. Different images have different structural features and spectral information and thus have different requirements for the amount of detail injection. Directly injecting details using the same gain can easily lead to too much injection to cause spectral distortion or insufficient injection to cause image blur. Therefore, it is necessary to retain the spectral and detail information through an injection amount coefficient *g*. After adding this coefficient, combined with formula (12), the new fusion image *F*_*k*_^(3)^ can be defined as follows:

(13) $$\begin{array}{c} F_{k}^{\left(3\right)}=\tilde{{MS}_{k}}+g*\frac{\tilde{{MS}_{k}}}{\left(1/N\right)\sum_{i=1}^{N}\tilde{{MS}_{k}}}.*D\text{.} \end{array}$$

We need to determine different coefficients *g* for remote sensing images with different structural features. The author in [38] believes that the requirements for spectral fidelity and spatial resolution in the injection model need to be balanced, and the weighted sum of the two evaluation indicators can be used as the fusion image indicator, and the weight indicates the degree of importance. We set the initial value *g*_0_ of the detail injection coefficient *g* and the step size *r*. For *g*_0_ ≤ *g* ≤ 1, the corresponding fused image is obtained and the weighted evaluation index is calculated and obtain the *g*_max_ that makes the evaluation index the highest as the final injection coefficient. For the evaluation index, this paper proposes a comprehensive evaluation system of spectral information and spatial information based on correlation analysis. The weighted evaluation index *Q* for spectral information and spatial information is defined as follows:

(14) $$\begin{array}{c} Q=\left(1-\alpha \right)*E_{SP}+\alpha *E_{HF}\text{,} \end{array}$$

where *E*_*SP*_ and *E*_HF_ represent the spectral information evaluation index and the spatial information evaluation index, respectively; *α* represents the weight of the two. For an image *F*_*k*_^(3)^ fused by formula (13), its *I* component is denoted as *I*_3_, then *E*_SP_ is represented by the average value of the correlation between the fused image and each channel *k* of the upsampled MS image, and *E*_*HF*_ is represented by the linear correlation between *I*_3_ and PAN images, which is defined as follows:

(15) $$\begin{array}{c} E_{SP}=\frac{1}{N}\sum_{k=1}^{N}corr\left(F_{k}^{\left(3\right)},{\tilde{MS}}_{k}\right)\text{,}\\ E_{HF}=corr\left(I_{3},P_{I}\right)\text{.} \end{array}$$

The authors in [28] show that the optimal determination of the injection components and the injection amount *g* follows the premise that if there is a higher correlation between *I* and PAN images, there will be a better fusion result. That is, if the correlation between the two is high, the spectral information can be well preserved when details are injected. At this time, the weight of the spatial feature can be increased and the weight of the spectral feature can be reduced. Conversely, if the correlation is low, the injected details are likely to cause spectral distortion. The spectral information weight should be increased, and the spatial information weight should be decreased.

Assuming that the *I* component corresponding to the fused image generated by the initial value *g*_0_ is denoted as *I*_0_, the weight *α* in formula (14) can be determined by the following formula:

(16) $$\begin{array}{c} \alpha ={\left(corr\left(I_{0},P_{I}\right)\right)}^{2}\text{.} \end{array}$$

The design of the square term of (16) has two reasons. The first is to avoid that the spectral weight is too small when *I*_0_ and *P*_*I*_ are highly correlated; the second is to magnify the difference between the spatial information of different images.

For different injection coefficients *g*, iteratively calculating *F*_*k*_^(3)^ according to formula (13), and calculating the evaluation index *Q* according to formula (14), *g*_max_ with the highest index *Q* can be obtained as the injection coefficient of the final fused image. *g*_max_ can be expressed as follows:

(17) $$\begin{array}{c} g_{\max}=\underset{g}{\arg}\max\;Q\left(F_{k}^{\left(3\right)}\left(g\right)\right)\text{.} \end{array}$$

Note that *g*_max_ can be different according to the characteristics of the image, such as the image dimension and the type of land cover. Combining formulas (13) and (17), after using the optimized injection coefficient, the final fused image can be updated as

(18) $$\begin{array}{c} F_{k}^{\left(3\right)}=\tilde{{MS}_{k}}+g_{\max}*\frac{\tilde{{MS}_{k}}}{\left(1/N\right)\overset{N}{\underset{i=1}{\sum }}\tilde{{MS}_{k}}}.*D\text{.} \end{array}$$

## 4. Analysis of Experiment Result

### 4.1. Experimental Setup

To evaluate the performance of our method, we use two datasets from the satellites, including QuickBird and IKONOS. These remote sensing images have different features in spectral wavelength, spatial resolution, etc., and all include four bands such as red, green, blue, and near-infrared.

For parameter setting, this paper sets the initial value of the injection volume coefficient *g*_0_ = 0.1 and the step size *r* = 0.05, to thoroughly consider the efficiency and precision of the calculation. The Gaussian filter window is set to 5 × 5, the default setting in reference [22].

This paper carried out two kinds of experiments. One is the simulated image after down-sampling the real image, also known as the reduced-scale (RS) experiment, that is, following the Wald protocol [39], and using the original MS image as a reference image. The simulated images contain a total of 100 sets of images from the datasets with 50 sets in each dataset. The other is the real image experiment, also known as the full-scale (FS) experiment. The real images contain the same number of images as the simulated images.

The methods used for comparison include classical and state-of-the-art methods such as bilateral filter luminance proportional (BFLP) [40], AWLP [9], context-based decision (CBD) [29], GLP-MTF [13], MF [12], adaptive spectral intensity modulation pansharpening (ASIMP) [41], and low-rank fuzzy fusion (LRFF) [42]. These methods are subjectively and objectively evaluated and are named qualitative and quantitative evaluations. The common objective evaluation indicators used with reference images are CC↑ [43], UIQI↑ [42], RMSE↓ [20], RASE↓ [16], SAM↓ [21], ERGAS↓ [44], and Q2n↑ (*Q*4/*Q*8) [44]. Commonly used objective metrics for real image fusion include *D*_*λ*_↓, *D*_*s*_↓, and QNR↑ [8]. ↑ means the optimal value is 1, while ↓ means the optimal value is 0. The details are shown in Table 1.

### 4.2. Performance Analysis

**Figure 2:**
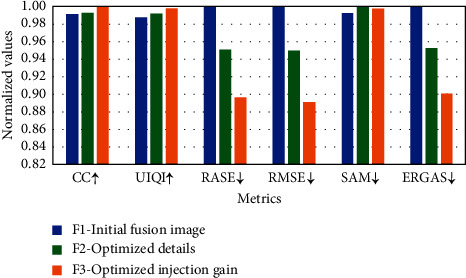
Average quantitative comparison of optimization steps.

To verify the effectiveness of the optimization method proposed in Sections 3.2 and 3.3 of this paper and quantitatively analyze the performance improvement achieved by the proposed method, we calculate the average objective indicators of each optimization step. The fused images corresponding to the three optimization steps are expressed as *F*1 representing the initial fusion image, *F*2 representing the image after Gaussian filtering to optimize the details, and *F*3 representing the image after adaptively optimizing the injected coefficients. The average objective index of 80 groups of image fusion results is calculated, respectively. To more intuitively show the relative changes of each indicator, each objective indicator is normalized, that is, for the result *X*(*i*) of an indicator *X*, the normalization is *X*(*i*)/(max (*X*(*i*))), the results are shown in Figure 2. We can see that for the initial fusion image *F*1, the injected details are obtained using the classical MRA-based method. After optimizing the details through the estimated Gaussian filter, the obtained details have a higher correlation with the MS image, and various indicators are significantly improved; after optimizing the injection coefficient, it can effectively prevent overinjection or underinjection, and all indicators are further improved. With the sequential optimization steps of *F*2 and *F*3, the overall indicators are getting better and better. Among them, the SAM index is basically stable. With optimization, the SAM index first increases slightly and then decreases slightly, showing that the proposed method has little impact on the loss of spectral information.

### 4.3. Simulation Experiment

In this paper, two sets of remote sensing images are used for subjective evaluation, and then the average objective evaluation is measured on 50 sets of images from each dataset.

**Figure 3:**
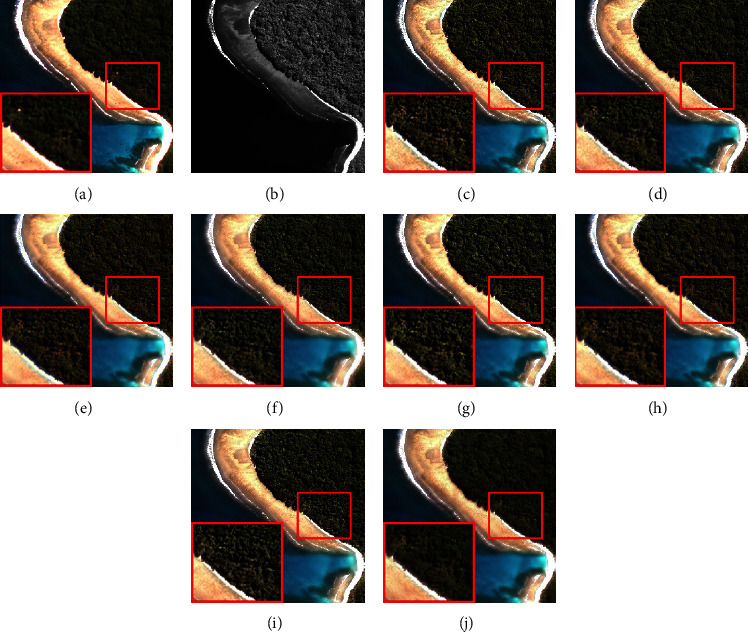
Fusion results of the images from QuickBird dataset in simulation experiment. (a) Reference. (b) PAN. (c) AWLP. (d) BFLP. (e) CBD. (f) MTF-GLP. (g) MF. (h) ASIMP. (i) LRFF. (j) Proposed.

**Figure 4:**
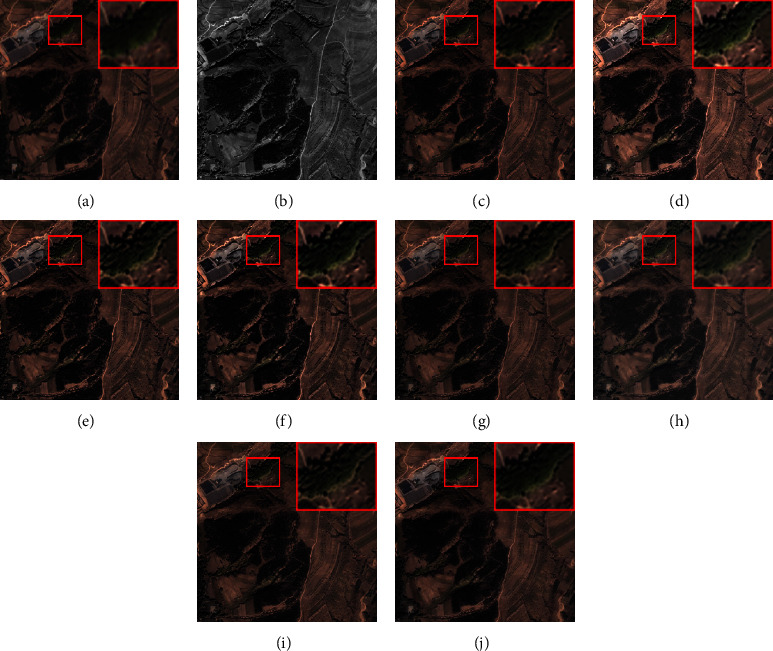
Fusion results of the images from IKONOS dataset in simulated experiment. (a) Reference. (b) PAN. (c) AWLP. (d) BFLP. (e) CBD. (f) MTF-GLP. (g) MF. (h) ASIMP. (i) LRFF. (j) Proposed.

The size of a simulation MS image is 256 × 256 × 4. The fusion results of each method on the QuickBird and IKONOS datasets are shown in Figures 3 and 4, respectively. The smaller red rectangle is enlarged and displayed in a larger rectangle. From Figure 3, we can see that the results of AWLP, CBD, MTF-GLP, and MF methods generate obvious spectral distortion in the forest area, and the color of them is brighter than the reference image; the results of BFLP and LRFF have slight spectral distortion, and there is an overinjection problem which introduces unnecessary noise. The result of ASIMP turns into brown in the forest area. The proposed method is relatively close to the reference image in the spectral information of the beach and forest. The fusion results in Figure 4 have exhibited a situation similar to those in Figure 3. The results of comparison methods show different degrees of spectral distortion, and the result of the proposed method is closest to the reference image.

To objectively evaluate the performance of each comparison method, the average objective metrics were calculated after the fusion of 50 sets of remote sensing images from each dataset. The results are shown in Table 2. We can see from the table that our method achieves the best results on all average metrics on both datasets.

### 4.4. Real Image Experiment

The experiment on real images uses a set of images from each dataset for subjective evaluation and calculates the average objective index for all sets of real images from the datasets.

**Figure 5:**
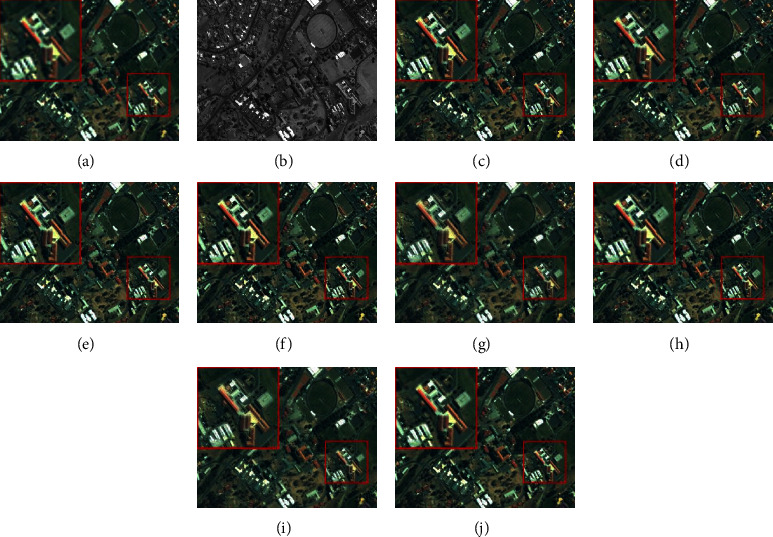
Fusion results of the images from IKONOS dataset in real experiment. (a) Up sampled MS. (b) PAN. (c) AWLP. (d) BFLP. (e) CBD. (f) MTF-GLP. (g) MF. (h) ASIMP. (i) LRFF. (j) Proposed.

**Figure 6:**
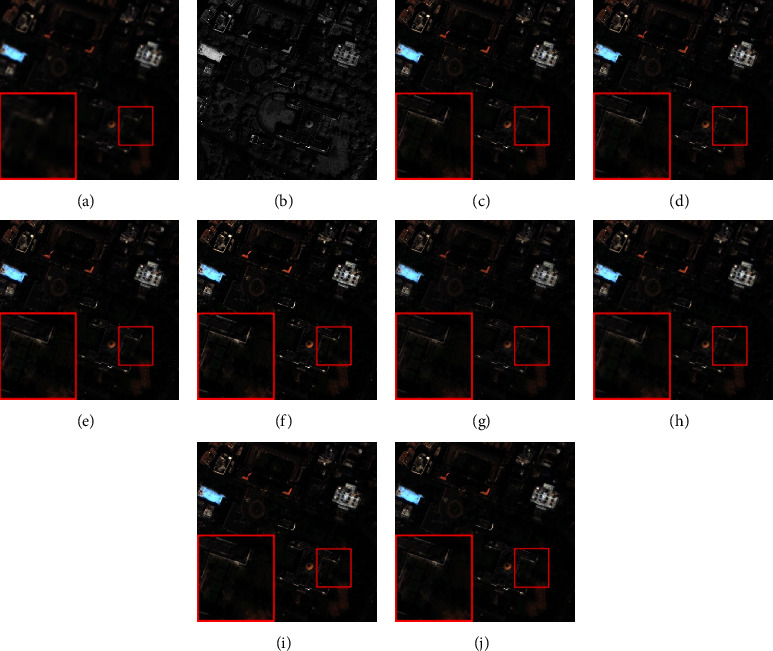
Fusion results of the images from QuickBird dataset in real experiment. (a) Up sampled MS. (b) PAN. (c) AWLP. (d) BFLP. (e) CBD. (f) MTF-GLP. (g) MF. (h) ASIMP. (i) LRFF. (j) Proposed.

The fusion results of the comparison methods on the IKONOS and QuickBird datasets are shown in Figures 5 and 6, respectively. From Figure 6, we can see that the results of the BFLP, MF, and LRFF methods produce apparent spectral distortion in the orange roof area. Since there is no reference image, it is difficult to directly distinguish other methods from the proposed method visually. We mainly refer to quantitative evaluation.

To objectively evaluate the effectiveness of each compared method, this paper conducts experiments on 50 sets of real images from each dataset and calculates the average objective indicators as shown in Table 3. The table shows that except for *D*_*s*_ in the QuickBird dataset, all the metrics of the proposed method are optimal, further verifying the good performance of our method.

## 5. Conclusion

In the fusion of remote sensing images, there has always been a key problem on how to keep the spectral information fidelity while injecting details. In this paper, a novel adaptive injection-based pansharpening method is proposed, which first uses the traditional injection model as the initial fusion image and then optimizes the injection details and injection gain. Gaussian filter estimation is used to simulate the characteristics of the MS sensor in the process of optimizing the injected details, and the PAN image is deconvolved with the estimated Gaussian filter to obtain the optimized details. To optimize the injection benefit, the spectral information and detailed information are comprehensively considered, and a weighted evaluation index is established to determine the adaptive injection amount coefficient. The fusion experiments are conducted on 100 sets of simulated images and real images from the IKONOS and QuickBird datasets. Compared to some advanced fusion methods, the qualitative and average quantitative evaluations show that our method performs better than all other comparison methods.

## Figures and Tables

**Table 1:** Illustration of the fusion quality metrics.

	Full name	Description	RS/FS	Optimal
CC↑	Correlation coefficient	Evaluating spatial similarity between the two images	RS	—
UIQI↑	Universal image quality index	Representing a global fusion quality	RS	1
RMSE↓	Root mean square error	Representing the difference between the two images	RS	0
RASE↓	Relative average spectral error	Reflecting the spectral error of the fusion image	RS	0
SAM↓	Spectral angle mapper	Measuring the spectral distortion	RS	0
ERGAS↓	Error relative global adimensionnelle de synthese	Evaluating the spatial and spectral quality	RS	0
*Q*4/*Q*8↑	*Q*4 for a 4-band image, *Q*8 for an 8-band image	A vector extension of the *Q*-index to evaluating the global quality	RS	1
*D*_*λ*_↓	—	Indicating spectral distortion	FS	0
*D*_*s*_↓	—	Indicating spatial distortion	FS	0
QNR↑	Quality with no reference	Comprehensive metric combining *D*_*λ*_ and *D*_*s*_	FS	1

**Table 2:** Average quantitative evaluation of fusion results in simulation experiments.

Methods	QuickBird dataset						IKONOS dataset					
	CC↑	UIQI↑	RASE↓	RMSE↓	SAM↓	ERGAS↓	CC↑	UIQI↑	RASE↓	RMSE↓	SAM↓	ERGAS↓
AWLP	0.9015	0.8567	21.3640	14.3177	3.7094	5.2526	0.9165	0.8934	20.9276	20.5598	4.0982	4.5009
BFLP	0.8964	0.8189	29.2416	18.9560	3.8910	6.9647	0.9137	0.8983	19.5769	20.1413	4.0510	4.8381
CBD	0.8943	0.8491	21.7795	14.8396	4.2800	5.5706	0.9177	0.8944	21.3869	22.0035	3.7171	5.8970
MTF-GLP	0.8959	0.8448	22.5773	15.4283	3.3850	5.6583	0.9188	0.9001	19.2471	19.8020	3.2134	4.8311
MF	0.9057	0.8643	18.2789	12.4872	3.8328	4.6707	0.9142	0.9002	17.3611	17.8616	4.1449	4.3381
ASIMP	0.9084	0.8627	19.9071	13.2617	3.8361	5.0637	0.9300	0.9043	16.8757	17.3623	3.5643	4.2421
LRFF	0.9078	0.8635	19.0474	13.0131	3.5951	4.9042	0.9266	0.9045	16.3265	17.5784	3.6155	4.2052
Proposed	**0.9125**	**0.8725**	**18.2357**	**12.3240**	**3.2945**	**4.6290**	**0.9315**	**0.9091**	**15.6355**	**16.0863**	**3.1610**	**3.9250**

The bold values indicate that they are optimal.

**Table 3:** Average quantitative evaluation of real experiments.

Methods	IKONOS dataset			QuickBird dataset		
	*D*_*λ*_↓	*D*_*s*_↓	QNR↑	*D*_*λ*_↓	*D*_*s*_↓	QNR↑
AWLP	0.0234	0.0481	0.9304	0.0446	0.0563	0.9021
BFLP	0.0230	0.0471	0.9318	0.0453	0.0578	0.9001
CBD	0.0259	0.0480	0.9281	0.0444	0.0608	0.8979
MTF-GLP	0.0295	0.0534	0.9194	0.0515	0.0601	0.8971
MF	0.0236	0.0523	0.9261	0.0489	0.0660	0.8892
ASIMP	0.0299	0.0528	0.9216	0.0405	**0.0531**	0.9091
LRFF	0.0338	0.0462	0.9211	0.3412	0.0573	0.9021
Proposed	**0.0188**	**0.0458**	**0.9370**	**0.0331**	0.0540	**0.9153**

The bold values indicate that they are optimal.

## Data Availability

The datasets can be obtained from https://www.kosmos-imagemall.com/.
